# Effects of co-treatment of *Plasmodium berghei*-infected mice with aqueous extract of *Ocimum gratissimum* leaves and primaquine on glucose-6-phosphate dehydrogenase activity, hematological, and antioxidant parameters

**DOI:** 10.1016/j.jaim.2022.100656

**Published:** 2022-11-16

**Authors:** Shedrach Kanu C, Chinyere Aloke, Nwabueze Elom I, Chinedum Eleazu O

**Affiliations:** a Department of Biochemistry, Alex-Ekwueme Federal University Ndufu-Alike, Ikwo, Ebonyi State, Nigeria; b Department of Medical Biochemistry, Faculty of Basic Medical Sciences, Alex Ekwueme Federal University, Ndufu-Alike, Ikwo, Abakaliki, Ebonyi State, Nigeria; c Protein Structure-Function and Research Unit, School of Molecular and Cell Biology, Faculty of Science, University of the Witwatersrand, Braamfontein, Johannesburg, 2050, South Africa; d Department of Chemistry, Alex-Ekwueme Federal University, Ndufu-Alike Ikwo, Ebonyi State, Nigeria

**Keywords:** Anti-malarials, Antioxidants, Erythrocytes, G6PD

## Abstract

**Background:**

It has been observed that most malaria patients especially G6PD-deficient patients usually experience oxidative stress and severe anemia when treated with primaquine. This calls for the need to search for a treatment option that will ameliorate these side effects.

**Objective:**

The effect of co-treatment of malaria with aqueous extract of *Ocimum gratissimum leaves* (AEOGL) and primaquine on G6PD activity, antioxidant indices and hematological parameters in *Plasmodium berghei*-infected mice was investigated.

**Materials and methods:**

Thirty mice divided into six groups of five mice each were recruited for this study. Whilst Group 1 (G1) served as the negative control (group not infected with plasmodium parasite), Groups 2 to 6 (G2-G6) were inoculated intraperitoneally with 0.2 ml of 1 × 10^5^/ml *Plasmodium berghei* (NK 65 strain) infected erythrocytes. G2 (parasite control) received no treatment. Groups 3,4,5 and 6 were administered 0.25 mg/kg bw of primaquine only; 100 mg/kg b. w of AEOGL +0.25 mg/kg bw of primaquine; 200 mg/kg b. w of AEOGL +0.25 mg/kg bw of primaquine; 200 mg/kg b. w of AEOGL respectively, for 14 days.

**Results:**

Treatment with only primaquine gave the highest mean malaria parasite clearance (82.10 ± 0.45 percent), followed by 100 mg/kg b. w of AEOGL + Primaquine (75.59 ± 0.47 percent), 200 mg/kg b. w of AEOGL + Primaquine (67.35 ± 0.67 percent), and AEOGL alone (55 ± 0.56 percent). In comparison with the untreated malaria groups, co-treatment with AEOGL + Primaquine produced a significant (p < 0.05) increase in G6PD activity, serum ascorbate, reduced glutathione, catalase activity, and a significant (p < 0.05) decrease in malondialdehyde level in a dose-dependent pattern and also a significant (p < 0.05) rise in packed cell volume, haemoglobin, and red blood cell count, unlike treatment with only primaquine which resulted in a non-significant (P > 0.05) difference in these parameters.

**Conclusion:**

Co-treatment of *Plasmodium berghei-*infected mice with AEOGL and primaquine improved the G6PD activity, hematological parameters and antioxidant status relative to treatment with only primaquine.

## 1 Introduction

Malaria is an ailment orchestrated by protozoan parasites belonging to the genus Plasmodium and is major cause of mortality and morbidity particularly in the tropical and sub-tropical regions of the world [1]. Annually, approximately 2 billion people are exposed to malaria whereas about 1.2 billion persons are vulnerable [2]. Malaria is the most common disease in tropical nations, with more than 219 million symptomatic cases and 435,000 estimated mortalities, according to latest data [2,3]. Due to the development of resistance, as well as high patronage and reliance on the efficacy of herbal therapies, there is a reliance on the practice of using herbal antimalarials in combination with conventional antimalarial drugs [4,5]. Some patients use this strategy to improve efficacy and compliance [6]. This method could be taken without considering the possibility of antagonistic effects from a herb–drug combination. According to the literature, this combination therapy is widespread in malaria-endemic nations where standard antimalarial medications are expensive and out of reach for most rural residents [7,8]. Most of anti-malaria medications, such as primaquine, exert their antiplasmodial effect while also releasing free radicals [9]. These free radicals are both friends and foes to the host metabolic processes—friends in the sense that they eliminate the parasite, but foes in the sense that they harm the cells and tissues of the host, causing cellular injury and inflammation, particularly in red blood cells and hepatocytes [10]. It has been reported that plasmodium infection affects G6PD activity [50,51]. Glucose-6-phosphate Dehydrogenase (G6PD) enzyme play important role in survival of erythrocytes. In Pentose phosphate pathway of erythrocytes, G6PD enzyme mediates the production of NADPH and GSH. GSH thus produced can react with H_2_O_2_ reducing it to water thereby preventing the generation of oxidative stress in the red blood cells. Unfortunately, cells deficient in G6PD enzymes are prone to oxidative stress when exposed to proxidants like primaquine [50]. Although primaquine is a potent orthodox antimalarial drug, there are cases of some side effects arising from its use in the treatment of G6PD-deficient and sickle cell patients suffering from malaria; side effects such as oxidative stress and severe anemia [11,45]. This calls for the need to search out a safer treatment regimen which in addition to their antimalarial effect, will also improve the antioxidant status and hematological indices of G6PD-deficient and sickle cell patients suffering from malaria, thereby protecting them from oxidative stress and anemia.

In quest of a better therapy alternative, phytochemicals derived from plants have been shown to be highly effective as anti-plasmodial medicines over time due to their antioxidant properties as well as their chemo-protective effects against diseases [12]. *Ocimum gratissimum (O. gratissimum)* belongs to the Psidium genus of the myrtle family (Myrtaceae). *O. gratissimum* leaves extract have been linked to anti-oxidant, anti-cancer, anti-plasmodial, anti-bacterial, anti-inflammatory, and anti-pain mechanisms [12,13]. In the preparation of soup, fish, meat, and stew, *O gratissimum* is used as a vegetable and a natural flavoring ingredient. Cough, pneumonia, fever, inflammation, cancer, anemia, diarrhoea, aches, fungal and bacterial infections are among the disorders for which it is used in folk medicine [14,15]. *In vitro* investigations have proven that *O. gratissimum* leaf extract has strong antioxidant properties [16]. Despite the use of *O. gratissimum* in folk medicine, there is paucity of information as regards their effect on the hematological and antioxidant indices of a plasmodium infected host when co-administered with orthodox antimalarial drugs. The goal of this study was therefore to see how co-treatment of malaria parasite with aqueous extract of *Ocimum gratissimum* leaves (AEOGL) and primaquine affects G6PD activity, hematological parameters and oxidative stress indices in *Plasmodium berghei* infected mice as against treatment with only primaquine. This will help patient and care givers in deciding the best and most suitable treatment regimen especially for oxidative stress and anaemia prone patients like sickle cell and G6PD-deficient patients.

## 2 Materials and Methods

### 2.1 Materials

#### 2.1.1 Chemicals and drugs.

Commercial ELISA test kits used for the assays were bought from Randox Laboratories Ltd., Crumlin, UK. Primaquine phosphate manufactured by IPCA, India was used as the standard drug. *Plasmodium berghei* NK 65 strain purchased from Nigeria Medical Research Institute Lagos was used to induce plasmodium infection. The rest of the chemicals and reagents utilized in this experiment were of analytical grade.

#### 2.1.2 Collection of plant materials and authentication

The leaves *of Ocimum gratissimum* were obtained at harvest from local farmers in Ebonyi State. Dr. Eric Nwancho of the Department of Plant Science at Ebonyi State University in Abakaliki, Nigeria, identified and validated the plant. The plant's voucher specimen number is EBSU/FBS/0097. The Departmental Herbarium received a sample of the plant component for future use.

### 2.2 Methods

#### 2.2.1 Aqueous extract of *Ocimum gratisimum* preparation

*O.gratissimum* leaves were air-dried at room temperature. An electric blender was used to grind the air-dried leaves into powder. The sample (50 g) was soaked for 24 h at room temperature in 1000 mL of de-ionised water, with periodic shaking. After that, it was filtered with Whatman filter paper (No.1). To get the aqueous crude extract, the filtrate was concentrated to dryness using a rotary evaporator at 45 °C, and the extract was preserved in the fridge at 4 °C until when used.

#### 2.2.2 Phytochemical analysis

Qualitative phytochemical screening for steroid, triterpenoid, alkaloid, flavonoids, ascorbate, polyphenols and tannins were done following the procedure described by Kujeke et al., [17]. Quantitative phytochemical analysis was done using the method described by Chukwuma et al. [18].

#### 2.2.3 Acute toxicity test of AEOGL

The median lethal dosage (LD 50) of AEOGL was calculated using Lorke's modified procedure [19]. For the LD test, thirteen mice weighing 30–35 g were acquired from the University of Nigeria, Nsukka's Veterinary Medicine Department. The mice were housed in normal cages and fed commercial rat chow and tap water ad libitum, with an average ambient temperature of 26 ± 1 °C and a 12 h light/12 h dark cycle. In the first stage, the animals were divided into three groups of three mice each and given graded oral dosages of O gratissimum extract dissolved in a mixture of dimethyl sulfoxide (DMSO) and H2O (0.5:9.5 v/v) at 10, 100, and 1000 mg/kg body weight, respectively. In the second stage, three mice were divided into three groups and given orally 1600, 2900, and 5000 mg/kg body weight of dissolved AEOGL, respectively, with one mouse serving as a control. For the lethal dose determination, the mice were examined for toxicity indications and possible death within 24 h of injection in each group. The LD of AEOGL was observed to be higher than 5000 mg/kg body weight, which was deemed safe, and dosages of 100 and 200 mg/kg body weight were chosen for the experiment based on this.

#### 2.2.4 Procedure for counting malaria parasite [28]

Immersion oil was placed on the film and viewed using ×100 objective. Between 10 and 20 fields were examined and average number of parasites per high power field (hpf) recorded. Average number of parasites per hpf was multiplied by 500. This gives the number of parasites per microliter of blood. The count was done in triplicate and the mean calculated.

#### 2.2.5 Formular for calculating percentage parasite clearance [28]

The percentage clearance of plasmodium parasite was done by the procedure outlined by Ref. [28] with a slight modification.

$$Mean\;\%\;parasite\;clearance=\frac{\text{Mean}\;\text{parasite}\;\text{count}\;\text{before}\;\text{treatment}-\text{Mean}\;\text{parasite}\;\text{count}\;\text{after}\;\text{treatment}}{\text{Mean}\;\text{parasite}\;\text{count}\;\text{before}\;\text{treatment}}\times 100$$

#### 2.2.6 Experimental animals

The Nigerian Institute of Medical Research in Lagos provided a total of thirty three weeks old male BALB/c mice weighing 30–35 g. They were allowed a seven-day acclimatization period before being used. The animals were kept in a plastic/wire gauze animal cage and fed normal diet with unlimited access to water.

#### 2.2.7 Ethical approval

All animal research were carried out according to a protocol approved by the Faculty of Biological Science Ndufu-alike, Ebonyi State, Nigeria's animal care and use committee with the ethical code AEFUNAI/FBS/00982.

#### 2.2.8 Experimental design

This research used thirty mice with a weight range of 30–35 g. Twenty-five mice were intraperitoneally injected with 0.2 ml of 1 × 10^5^/ml Plasmodium berghei (NK 65 strain) parasitized erythrocytes and randomly divided into five (G2-G6) groups, each with five mice, while the remaining five mice (G1) served as the normal control (group not infected with parasite). All mice in the respective groups were treated daily for fourteen days as follows:

Group 1: Given 2 ml distilled water daily as negative control group (mice not infected with plasmodium).
Group 2: Given 2 ml distilled water only without treatment (disease control)
Group 3: Orally administered 1 ml of 0.25 mg/kg b. w. primaquine +1 ml of distilled water daily
Group 4: Orally administered 1 ml of 100 mg/kg b. w. AEOGL + 1 ml of 0.25 mg/kg b. w. primaquine daily
Group 5: Orally administered 1 ml of 200 mg/kg b. w. AEOGL + 1 ml of 0.25 mg/kg b. w. primaquine daily
Group 6: Orally administered 200 mg/kg b. w. of AEOGL daily +1 ml of distilled water daily

The animals were kept at a room temperature of 24 °C with alternate 12-h light and dark cycle.

### 2.3 Sample collection and processing

On the last day of the experiment (day 15), the animals were weighed using a sensitive balance. They were anaesthesized by inhalation of formaldehyde and bled by cardiac puncture after which the animals were sacrificed. Blood sample was collected in plain tubes and also in anticoagulant tubes. The blood in the plain tubes were centrifuged (3000 g for 15 min) for the separation of serum. The serum was used for biochemical assays. Thick and thin blood film were immediately prepared directly from the whole blood while bleeding.

### 2.4 Determination of biochemical parameters

#### 2.4.1 Glucose-6-phosphate dehydrogenase (G6PD) activity

The activity of G6PD in anticoagulated blood was assayed following the method of Konberg & Horecker [20] described in Randox Laboratories assay kit for G6PD activity.

#### 2.4.2 Assay of Catalase activity

The activity of calatase (CAT) in serum was measured using Machly and Chance's procedure [21]. Catalase breaks down H2O2 into H2O and O2. After 10 min, the concentration of H2O2 was measured using a spectrophotometer, and the catalase activity was calculated in units/mg protein. At 230 nm, the absorbance was measured.

#### 2.4.3 Assay of superoxide dismutase (SOD) activity

The method published by Martin et al. [22] was used to conduct the serum SOD activity assay. Precisely, 920 μL of 0.05 M assay buffer (Phosphate buffer pH 7.8) was introduced to a clean test tube containing 40 μL of sample, stirred, and incubated at 25 °C for 2 min. After that, 40 μL of hematoxylin solution was then added, immediately mixed, and the absorbance at 560 nm was determined. SOD inhibits auto-oxidation of hematoxylin at the test pH, and the percentage inhibition is related to the amount of SOD contained within a given range [23].

#### 2.4.4 Determination of Reduced glutathione concentration

In order to estimate the level of reduced glutathione in serum, the method published by Rukkumani et al. [24] was followed. In most cases, the reduced form of glutathione contains the majority of cellular non-protein sulfhydryl groups. This approach is based on the production of a fairly stable yellow color when sulfhydryl compounds are treated with 5, 5 – dithiobis – (2-nitrobenzoic acid) (Ellaman's reagent). Ellaman's reagent reacts with reduced glutathione to produce 2-nitro-5-thiobenzoic acid, a chromophoric compound having a molar absorption of 412 nm.

#### 2.4.5 Estimation of serum ascorbate

The serum ascorbate level was determined using 2, 4 – dinitrophenyl hydrazine method described by Teitz [25].

#### 2.4.6 Determination of thiobarbituric acid reactive substance (TBARS) concentration in serum

Fraga et al. [26] procedure was followed in determining the amount of thiobarbituric acid reactive substance (TBARS) in tissues. Malondialdehyde (MDA) interacts with thiobarbituric acid (TBA) at a low pH of 3.5 and a high temperature of 100 °C to generate a pink color that can be detected at 532 nm.

### 2.5 Assessment of hematological parameters

The approach outlined by Ochei et al. [27] was used to measure Pack Cell Volume (PCV), Hemoglobin Concentration (HB), and Red Blood Cell Count (RBC) using anticoagulated blood. Cheesbough's hemocytometry method [28] was used to determine total white blood cell counts (WBC), lymphocyte count, and eosinophil count.

### 2.6 Statistical analysis

The replicate results were analysed and presented as Mean Standard Deviation, with one-way analysis of variance (ANOVA) and Tukey's test used to determine significance at p < 0.05.

## 3 Results

### 3.1 Effect of Co-treatment with AEOGL and primaquine on mean percentage clearance of *Plasmodium berghei* in mice

The result (Table 2) below showed that the higher the dosage of AEOGL extract co-administered with the primaquine, the lower the percentage parasite clearance is inversely related to the percentage clearance of *Plasmodium berghei* in the mice. However, the group treated with only primaquine had a greater percentage of malaria clearance compared to the AEOGL extract + primaquine treated group.

**Table 1:** Result of qualitatitive and quantitative phytochemical analysis of aqueous extract of *ocimum gratissimum* leaf. As presented in Table 1 below, the phytochemical analysis results of aqueous extract of *O. gratissimum* leaf show that it is very rich in polyphenol, flavonoids, alkaloids, phytosterol and ascorbic acid.

Phytochemical	Total phytosterol	polyphenol	alkaloid	tanins	flavonoids	saponins	ascorbate
qualitative	+	++	++	+	++	+	++
quantitative	0.67 mg ± 2.35 mg/g	4.55 ± 1.39 mg/g GAE/g	2.56 ± 2.40 mg/g	0.45 ± 0.45 mg/g TAE/g	0.77 ± 1.95 mg/g QE/g	0.89 ± 1.98 mg/g	15.50 ± 0.43 mg/g

+ = Present; ++ = highly present; TAE = tanic acid equivalent; GA = Galic acid equivalent; QE = Quercitin Equivalent.

**Table 2:** Mean percentage clearance of *Plasmodium berghei* in mice.

Groups	Negative Control	Parasitic Control	0.25 mg/kg primaquine	100 mg/kg AEOGL +0.25 mg/kg primaquine	200 mg/kg AEOGL+ 0.25 mg/kg primaquine	200 mg/kg AEOGL
Percentage clearance (%)	0.00	0.00	82.10 ± 0.45	75.59 ± 0.47	67.35 ± 0.67^c^	55.0 ± 0.56^c^

The results are stated as Mean ± SD. values with superscript ‘a’ are significant at p < 0.05 when compared to the normal control group (group not infected with plasmodium). values with superscript ‘b’ are significant at p < 0.05 when compared to the parasite control group, whereas superscript c’ shows significant when compared to treatment with only primaquine. PQ = primaquine, AEOGL = Aqueous extract of *Ocimum gratissimum* leaf.

**Table 3:** G6PD enzyme activity of the various treatment groups.

Groups	Negative Control	Parasitic Control	0.25 mg/kg primaquine	100 mg/kg AEOGL +0.25 mg/kg primaquine	200 mg/kg AEOGL+ 0.25 mg/kg primaquine	200 mg/kg AEOGL
G6PD Activity (mU/g Hb)	6.15 ± 0.65	4.54 ± 0.55^a^	4.03 ± 0.34	5.77 ± 0.45^bc^	6.56 ± 0.33^bc^	5.45 ± 56

The results are stated as Mean ± SD. Bars with superscript ‘a’ are significant at p < 0.05 when compared to the normal control group (group not infected with plasmodium). Values with superscript ‘b’ are significant at p < 0.05 when compared to the parasite control group, whereas ‘c’ is significant at p < 0.05 when compared to only PQ treated group (GP 3). PQ = primaquine, AEOGL = Aqueous extract of *Ocimumgratissimum* leaf, U/mg = units per mg of enzyme proteins.

### 3.2 Effect of Co-treatment with AEOGL and primaquine on G6PD enzyme activity of *Plasmodium berghei* infected mice

There was a non-significant (p > 0.05) decrease in the G6PD enzyme activity of the group treated with only primaquine (GP3) compared to the parasite control group (see Table 3). However, aqueous extract + primaquine treatment significantly (p < 0.05) increased the activity of G6PD enzyme in a dose-dependent pattern compared to both the parasite control group and the group treated with only primaquine (GP3). Treatment with only AEOGL showed a non-significant increase compared to the parasite control group.

### 3.3 Effect of Co-treatment with AEOGL and primaquine on oxidative stress indices of *Plasmodium berghei* infected mice

There was a significant (p < 0.05) decrease in serum CAT, SOD, GSH, and ascorbate with a corresponding rise in MDA of the parasite control group compared to the negative control group (see Table 4 below). Co-treatment with AEOGL extract + primaquine produced a significant (p < 0.05) increase in serum CAT, SOD, GSH, and ascorbate with an attendant decrease in MDA compared to the parasite control (PC) group. However, the group treated with only primaquine did not show any statistically significant (p > 0.05) difference in these parameters as against the parasite control (PC) group.

**Table 4:** Oxidative stress indices of the various treatment groups.

Groups	Negative Control	Parasitic Control	0.25 mg/kg primaquine	100 mg/kg AEOGL +0.25 mg/kg primaquine	200 mg/kg AEOGL+ 0.25 mg/kg primaquine	200 mg/kg AEOGL
CAT (U/mg)	3.59 ± 0.34	2.61 ± 0.11^a^	2.45 ± 0.05	4.03 ± 0.02^b^	4.54 ± 0.01^b^	5.77 ± 0.04^b^
SOD (U/mg)	11.66 ± 0.02	11.00 ± 0.07	11.41 ± 0.03	11.27 ± 0.09	11.60 ± 0.05	12.99 ± 0.19^b^
GSH (μg/ml)	31.12 ± 0.07	28.63 ± 0.04^a^	29.97 ± 0.43	31.75 ± 0.29^b^	32.70 ± 0.05^b^	33.50 ± 0.38^b^
VIT C (mg/dl)	1.178 ± 0.03	0.74 ± 0.08^a^	0.73 ± 0.10	1.13±0–05^b^	1.12 ± 0.02^b^	2.15 ± 0.02^b^
MDA mg/dl	2.69 ± 0.52	4.70 ± 0.35^a^	4.33 ± 0.23	2.94 ± 0.24^b^	2.86 ± 0.23^b^	2.75 ± 0.23^b^

The results are stated as Mean ± SD. Bars with superscript ‘a’ are significant at p < 0.05 when compared to the normal control group (group not infected with plasmodium). Values with superscript ‘b’ are significant at p < 0.05 when compared to the parasite control group, whereas ‘c’ is significant at p < 0.05 when compared to only PQ treated group (GP 3) PQ = primaquine, AEOGL = Aqueous extract of Ocimumgratissimum leaf, U/mg = units per mg of enzyme proteins.

### 3.4 Effect of Co-treatment with AEOGL and primaquine on hematological indices of *plasmodium berghei*-infected mice

When compared to the negative control, a substantial (p < 0.05) decrease in PCV, HB, and RBC levels was observed in parasite control groups (see Table 5). In comparison with the parasite control group, co-treatment with AEOGL extract + primaquine resulted in a dose-dependent substantial (p < 0.05) rise in PCV, HB, and RBC levels, whereas primaquine alone resulted in a non-significant (p > 0.05) drop in these same parameters.

### 3.5 Effect of Co-treatment with AEOGL and primaquine on some immunological indices of *plasmodium berghei*-infected mice

The white blood cell count of all groups did not differ significantly (p > 0.05) (see Table 6). When compared to the negative control, only the parasite control group demonstrated a significant (p < 0.05) rise in eosinophils. The eosinophil count was significantly reduced after treatment with primaquine alone or a combination of primaquine and AEOGL.

## 4 Discussion

Malaria has long been a serious public health concern in Sub-Saharan Africa, causing extensive hematological and biochemical changes. Plasmodium-caused illness is frequently accompanied by significant oxidative damage and anemia [29]. In collaboration with the finding of Ihekwereme et al. [30], we noticed a dose depenedent antagonistic effect of *O. gratissimum* on Primaquine, which means that the higher the dose of *O. gratissimum* administered with primaquine, the lower the malaria parasite clearance. Being that primaquine exerts its antiplamodial effect via free radical attack [31], the high content of antioxidants present in *O. gratisimum* might be responsible for the reduced plasmodium parasite clearance of primaquine at higher dose of *O. gratissimum.*

G6PD's main physiological function is to produce NADPH and ribose 5-phosphate. NADPH regenerates reduced glutathione in erythrocytes, preventing hemoglobin denaturation, preserving the sulfhydryl groups on red blood cell membranes, and detoxifying hydrogen peroxide and oxygen radicals in and on red blood cells [31]. Reduced G6PD activity will result in low levels of NADPH, reduced-glutathion and ribose-5-phosphate as the enzyme's by-products. Overall, the host experiences lower antioxidant levels and higher oxidative stress as a result of this depletion. Because red blood cells are oxygen carriers and do not have mitochondria, they are the cells most severely affected by this condition. Hemolysis and anemia ensue as a result [50]. The low G6PD activity of the parasite control group as well as the primaquine treated group as found in this study is indicative of severe oxidative stress orchestrated by the pathophysiology of the parasitic infection [33] as well as the pro-oxidant effect of the drug [31]. However, the increased G6PD activity among the groups co-treated with primaquine and AEOGL extract portrays a therapeutic action with increased antioxidant activity (see Table 3). This treatment regimen is suggestive of a more beneficial therapeutic option for G6PD-deficient or sickle cell patients who are prone to oxidative stress and anemia.

Antioxidant enzymes like superoxide dismutase (SOD) and catalase (CAT), as well as serum ascorbate and reduced-glutathione, protect the biological system from oxidative stress by maintaining a normal level of free radicals [[34], [36]]. The fact that both the parasite control group and the group treated with only primaquine showed a decrease antioxidant level confirms oxidative stress [9,10,[35], [37]]. In contrast, the high antioxidant content of AEOGL (Polyphenols, ascorbic acid and flavonoids) and the complementary action of the bioactive compounds in the extract, which must have ameliorated the generation of reactive oxygen species and provided reducing equivalent to the enzymes [39], could have contributed to the increased antioxidant enzyme activities of the groups co-treated with primaquine and AEOGL. Similar findings have been reported in other studies [23,34,38]. Serum malondialdehyde (MDA) has been identified as a biomarker of lipid peroxidation caused by the attack of free radicals on the lipid membranes of macromolecules [40]. The increase of free radicals produces lipid peroxidation (measured as MDA), a process that causes catabolism of cell membrane lipids, resulting in organ damage, most likely due to the breakdown of polyunsaturated fatty acids in the liver, kidney, and red blood cells [41]. The increased rate of lipid peroxidation caused by increased free radical generation in the process of drug biotransformation and pathophysiology of plasmodium infection must have been responsible for the increased MDA level of the group treated with only primaquine as well as the parasite control group. In contrast, the reasonable reduction in serum MDA levels of the group co-treated with primaquine and AEOGL could be attributed to the antioxidants (ascorbic acid, flavonoids, and polyphenols) present in AEOGL. These antioxidants must have mitigated lipid peroxidation in the AEOGL-primaquine co-treated groups. The results of this investigation collaborates prior research findings [38,41,42].

Hematological markers for assessing anemia include pack cell volume (PCV), hemoglobin concentration (HB), and red blood cell count (RBC) [43]. Our results (Table 5) demonstrated a substantial drop in PCV, HB, and RBC of the parasite control group compared to the normal control group, which is consistent with other research [41,44,45]. When compared to the parasite control group, treatment with only primaquine resulted in a non-significant decrease in PCV, HB, and RBC. A proposed cause of this decline is membrane peroxidation caused by free radicals generated during drug metabolism [46]. However, the groups co-treated with primaquine and AEOGL extract showed a substantial increase in these parameters. The combined impact of the numerous antioxidants present in the extract, such as flavonoids, tannins, and alkaloids, might have been responsible for mopping up free radicals and protecting red cell membranes from lipid peroxidation and hemolysis [41].

**Table 5:** Hematological indices of the various treatment groups.

Groups	Negative Control	Parasitic Control	0.25 mg/kg primaquine	100 mg/kg AEOGL +0.25 mg/kg primaquine	200 mg/kg AEOGL+ 0.25 mg/kg primaquine	200 mg/kg AEOGL
PCV (%)	46 ± 1.07	30 ± 1.76^a^	33 ± 1.56	39 ± 2.56^bc^	45 ± 1.34 ^bc^	33 ± 1.45
HB (mg/dl)	15.33 ± 2.98	10.66 ± 0.89^a^	11.34 ± 1.78	12.66 ± 1.67^b^	15.60 ± 1.56^bc^	11.43 ± 0.54
RBC (X10^12^/L)	8.94 ± 0.35	4.54 ± 0.21^a^	5.30 ± 0.38	7.98 ± 0.18^b^	8.00 ± 0.02^b^	5.95 ± 0.46^b^

The results are stated as Mean ± SD. Bars with superscript ‘a’ are significant at p < 0.05 when compared to the normal control group (group not infected with plasmodium). Values with superscript ‘b’ are significant at p < 0.05 when compared to the parasite control group, whereas ‘c’ is significant at p < 0.05 when compared to only PQ treated group (GP 3) PQ = primaquine, AEOGL = Aqueous extract of *Ocimum gratissimum* leaf.

**Table 6:** Immunological indices of the various treatment groups.

Groups	Negative Control	Parasitic Control	0.25 mg/kg primaquine	100 mg/kg AEOGL +0.25 mg/kg primaquine	200 mg/kg AEOGL+ 0.25 mg/kg primaquine	200 mg/kg AEOGL
WBC (x10^9^/L)	9.45 ± 0.40	9.80 ± 0.25	9.60 ± 0.59	9.79 ± 0.16	9.68 ± 0.11^b^	9.10 ± 0.30^b^
Lymphocytes (%)	30.56 ± 1.67	25.67 ± 2.06	27.56 ± 1.78	33.45 ± 0.23	30.89 ± 0.87	25.67 ± 1.68
Eosinophil (%)	1.25 ± 0.25	7.00 ± 0.40^a^	1.75 ± 0.47^b^	2.50 ± 0.29^b^	1.35 ± 1.11^b^	3.50 ± 0.41^b^
Neutrophils (%)	67.25 ± 1.38	68.75 ± 0.63	67.5 ± 2.63	61.5 ± 4.64	59.50 ± 1.12	58.75 ± 0.75^b^
Basophil (%)	0.1 ± 0.02	0.2 ± 0.05	0.5 ± 0.03	1.03 ± 0.05	0.3 ± 0.01	0.5 ± 0.05

The results are stated as Mean ± SD. Bars with superscript ‘a’ are significant at p < 0.05 when compared to the normal control group (group not infected with plasmodium). Values with superscript ‘b’ are significant at p < 0.05 when compared to the parasite control group, whereas ‘c’ is significant at p < 0.05 when compared to only PQ treated group (GP 3) PQ = primaquine, AEOGL = Aqueous extract of *Ocimumgratissimum* leaf.

White blood cell (WBC) total and differential counts have been demonstrated in studies to be markers for determining the severity of infection [47,32]. Although total WBC and lymphocytes did not differ significantly between the parasite control and normal control groups, the eosinophil count of the parasite control group was significantly higher. This increase in eosinophils in relation to malaria infection supports Kurtzhals et al. [48] and Kotepui et al. [49] findings. Surprisingly, the treated groups (G3-G6) all showed lower eosinophil counts than the parasite control group (see Table 6). This demonstrates the effectiveness of combining AEOGL and primaquine to treat malaria parasite infection.

## 5 Conclusion

The outcome of this study demonstrated that co-treatment with the orthodox drug, primaquine and AEOGL improved G6PD activity, hematological and antioxidant indices as against single treatment with primaquine. This suggests that AEOGL had an antioxidant impact when co-administered with primaquine and could be a candidate for the development of an antimalarial-medicinal plant combination regimen to treat malaria especially in sickle cell and G6PD-deficient patients prone to oxidative stress.

### 5.1 Recommendation for further studies

This study could be stepped up further by using G6PD-deficient mice as the subjects.

## Data availability

If requested, all data will be made available.

## Authors’ contribution

Concept development: SCK. Experimental work: SCK. Data analysis: SCK and CA. Data Interpretation: SCK, CEO, and INE Writing: SCK, CEO, CA, NEI. The final manuscript was read and approved by all the authors.

## Funding

There was no external funding nor grant. All the authors contributed to the cost of this study.

## Declaration of competing interest

The authors declare no competing interest.
